# Multicellular ovarian cancer spheroids: novel 3D model to mimic tumour complexity

**DOI:** 10.1038/s41598-024-73680-6

**Published:** 2024-10-09

**Authors:** Inken Flörkemeier, Lisa K. Antons, Jörg P. Weimer, Nina Hedemann, Christoph Rogmans, Sandra Krüger, Regina Scherließ, Astrid Dempfle, Norbert Arnold, Nicolai Maass, Dirk O. Bauerschlag

**Affiliations:** 1 Department of Gynaecology and Obstetrics, University and University Medical Center Schleswig-Holstein Campus Kiel, Kiel, Germany; 2 Department of Pathology, University and University Medical Center Schleswig-Holstein Campus Kiel, Kiel, Germany; 3 Department of Pharmaceutics and Biopharmaceutics, Kiel University, Kiel, Germany; 4 KiNSIS Priority Research Area, Kiel University, Kiel, Germany; 5 Institute of Medical Informatics and Statistics, University and University Medical Center Schleswig-Holstein Campus Kiel, Kiel, Germany; 6 Department of Gynaecology, Jena University Hospital, Jena, Germany

**Keywords:** Ovarian cancer and fibroblast, Spheroids, Co-culture model, Tumour microenvironment, Cancer microenvironment, Cancer models

## Abstract

*In vitro*, spheroid models have become well established in cancer research because they can better mimic certain characteristics of *in vivo* tumours. However, interaction with the tumour microenvironment, such as cancer-associated fibroblasts, plays a key role in tumour progression. We initially focused on the interaction of tumour cells with fibroblasts. To model this interaction, we developed a spheroid model of ovarian cancer and fibroblasts. To this end, ovarian cancer cell lines and *ex vivo* primary cells were simultaneously and sequentially seeded with fibroblasts in a scaffold-free system at different ratios and subsequently characterized with respect to changes in morphology, proliferation, and viability. We demonstrated that co-cultures are able to form by far more compact spheroids, especially in cells that form aggregates in mono-culture. In addition, the co-cultures were able to increase proliferation and sensitivity to cisplatin. Simultaneous seeding led fibroblasts invade the core in both cell lines and primary cells. These results show differences in formation, firmness, and size between co-culture and mono-culture. Our model is designed to better represent and characterize the mutual influencing factors of fibroblasts and tumour cells. Fibroblast-supplemented multicellular spheroids are a valuable tool for tumour microenvironment interaction and new drug discovery.

**Supplementary Information:**

The online version contains supplementary material available at 10.1038/s41598-024-73680-6.

## Introduction

Ovarian cancer is currently the fifth leading cause of cancer-related death among women, and approximately 315,000 women globally are newly diagnosed with ovarian cancer each year^1,2^. Due to the lack of specific symptoms, the majority of patients with ovarian cancer are still being diagnosed at an advanced stage, leading to an unfavourable prognosis and poor 5-year overall survival^2^. Ovarian cancer encompasses a heterogeneous group including different histological and genomic profiles^3,4^. Standard of care first line treatment of ovarian cancer consists of surgical cytoreduction followed by platinumtaxane chemotherapy, with or without the angiogenesis inhibitor bevacizumab. In certain cases this treatment is followed by maintenance treatment with poly-ADP-ribose polymerase (PARP) inhibitors (PARPi)^5–8^. However, micrometastases often remain and become resistant to therapy, eventually leading to disease recurrence^9^.

Advanced ovarian cancer is characterized by metastases in the peritoneal cavity. Peritoneal carcinomatosis and malignant ascites, are frequently associated with advanced ovarian cancer^10^. Metastasis of ovarian cancer is predominantly routed transcoelomic. In this process, cancer cells are passively shed from the primary tumour into the peritoneal cavity (cell detachment) and subsequently transported via the ascites (dissemination), forming multifocal metastases (implantation)^11,12^. Beside tumour cells, ascites and solid tumours contain non-malignant cells and soluble factors promoting proliferation, dissemination and seeding of ovarian cancer spheroids in the peritoneal cavity^12^. Currently, it is reported that the tumour microenvironment (TME) plays a crucial role in ovarian cancer tumourigenesis^13,14^.

The TME generally consists of irregular formed tumour vessels, extracellular matrix, cancer-associated fibroblasts (CAF), and immune cells^12–14^. It interacts with cancer cells via paracrine signalling and cell-to-cell contact mechanisms, allowing growth factors, cytokines and chemokines, extracellular matrix proteins and extracellular vesicles to promote tumour formation^15^. The proportion of cancer cells and non-cancer cells differ interindividually and intraindividually during disease progression. Previous literature demonstrates a crucial impact of fibroblasts and CAFs in proliferation and the initial steps of ovarian cancer metastasis by secreting cytokines and chemokines^16^. Despite this importance of CAFs in tumour behaviour, little is currently known about how cancer cells convert normal fibroblasts into CAFs. Differences between the active CAF and normal fibroblasts are, in addition to the proximity to the tumour, mainly in the proliferation and metabolic capacity^17^. Through the secretion of soluble factors, CAFs actively promote tumour progression which stimulates survival, proliferation, and invasion. CAFs express various markers including smooth muscle α-actin, fibroblast activation protein (FAP), integrin β1, and several others. Thus, the frequency of CAFs is increased in the ascites and tumour of high-grade serous ovarian cancer compared to low-grade serous ovarian cancer^18^. In addition, CAFs were found to be increased in the metastatic omentum^19^. Paracrine signalling between cancer cells and fibroblasts showed mutual stimulation of proliferation and induction of drug resistance. Moreover, ascites is shown to form heterotypic spheroids consisting of a core of CAFs surrounded by tumour cells with high integrin-α5 expression^20^. Therefore, in the development and analysis of new tumour models, consideration of the microenvironment is essential to better represent the *in vivo* situation and to better assess the long-term effects of anti-cancer drugs.

Two-dimensional (2D) cell cultures are convenient and simple, but have significant limitations in reproducing the complexity and characteristics of naïve tumours^21^. A recognized model to study new drugs is nowadays the use of three-dimensional (3D) spheroid models^22–27^. In recent years, there have been numerous ideas to improve the development of *in vitro* tumour models. One approach is to create 3D multicellular tumour spheroids with physiological properties that are similar to *in vivo* tumour tissue and TME for efficient drug screening and successful drug development^28–30^. Traditional spheroid culturing using exclusively tumour cells is now extended to co-culturing cancer cells with fibroblasts. *In vivo*-like properties were observed for co-culture models, strongly supporting their usefulness as preclinical tumour models for drug screening and TME interactions^31,32^. The establishment of a new co-culture model for ovarian cancer creates future opportunities to investigate whether the presence of a complex microenvironment alters the sensitivity of cancer cells to chemotherapy and whether resistance to treatment observed *in vivo* can be better mimicked better and analysed with a multicellular model^14^. Overall, a new model should aim to provide a relevant and easy-to-use tool that can be readily introduced into routine preclinical screening of therapeutic strategies for the treatment of ovarian cancer. Improved 3D cell models also offer new perspectives for basic research, cancer research and the development of new drugs that could help to at least reduce the use of animals.

**Fig. 1:**
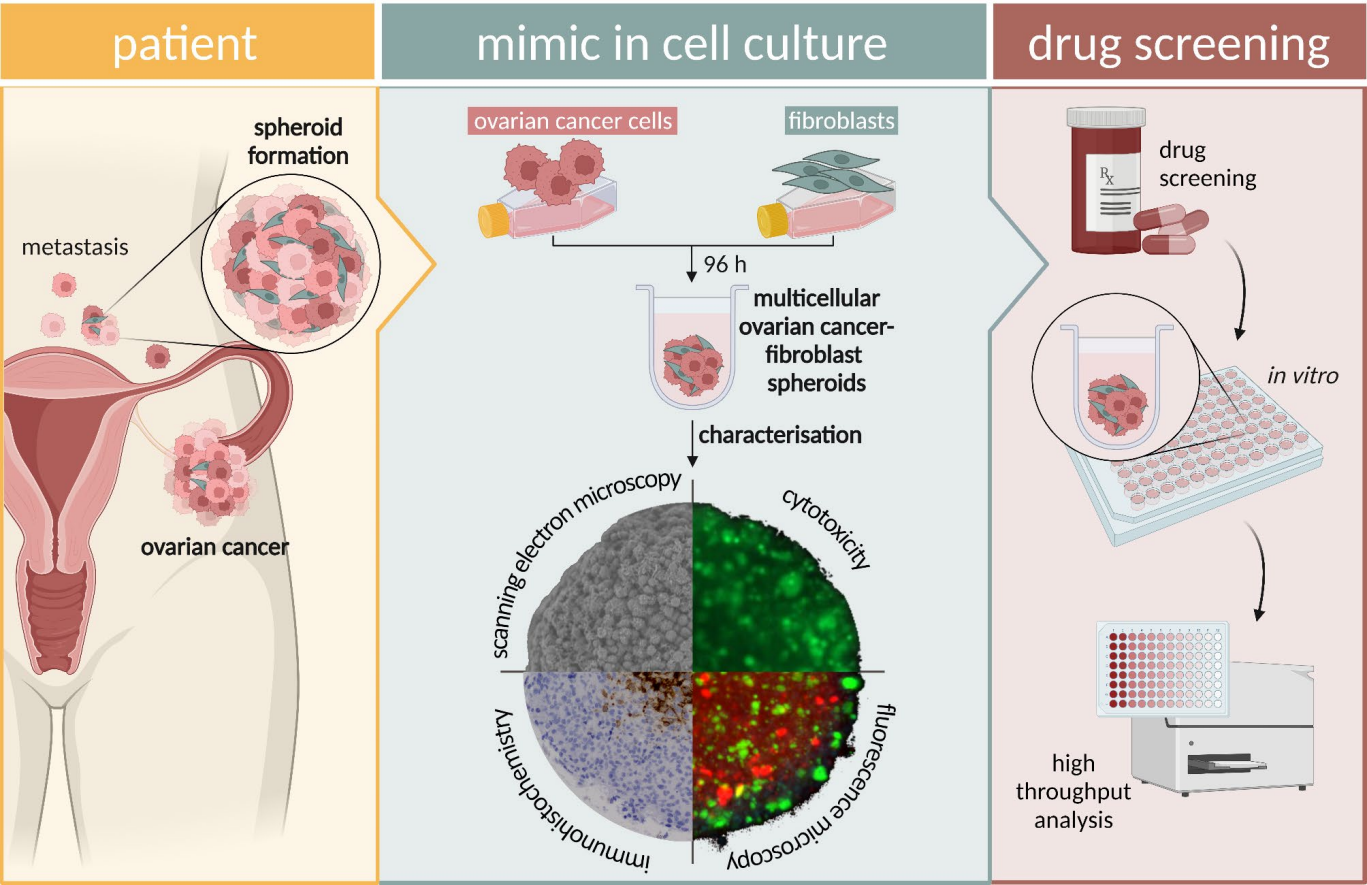
**Conceptual representation of the multicellular ovarian cancer-fibroblast spheroid model.** In the *in vivo* setting of ovarian cancer, the TME, including fibroblasts, has a significant impact on tumour characteristics and fibroblasts play an important role in both primary tumour formation and metastasis. CAFs play a crucial role, especially in the formation of heterotypic spheroids. To incorporate this into a model, a spheroid culture of ovarian cancer cells and fibroblasts was developed. For this purpose, cells were co-cultivated for 96 h in ultra-low attachment plates and characterised by scanning electronic microscopy, immunohistochemistry, fluorescence microscope and cytotoxicity assays. Systematic characterization of the generated spheroids (cell viability, spheroid geometry and morphology) is essential to ensure the suitability for drug screening.

In this study, we designed and characterised a multicellular 3D *in vitro* ovarian cancer spheroid model in which tumour cells are co-cultured and interact with fibroblasts (Fig. 1). We focused on characterising the morphology, proliferation and viability of the spheroids established in this model. In the present work, we compared simultaneous and sequential seeding of tumour cells and fibroblasts to develop a better model representing the microenvironment and ovarian cancer.

## Results

**Fig. 2:**
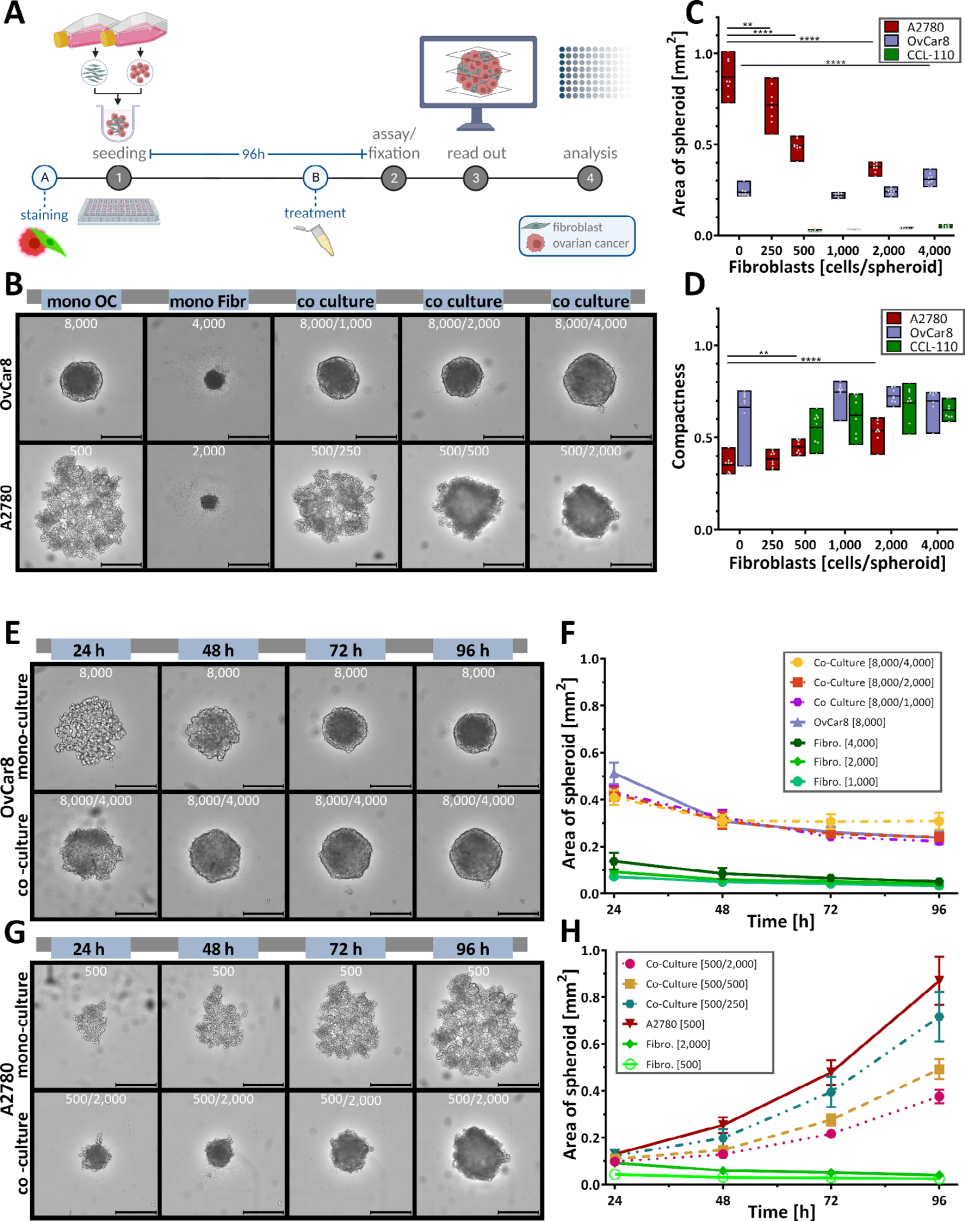
**Growth progression and growth kinetics of simultaneously seeded mono and co-cultured spheroids. **(**A**) Schematic creation of the co-cultivated spheroids, including tumour cells and human fibroblasts. Spheroids were cultured in mono-culture and in co-culture with ovarian cancer cells (OC) and different cell numbers of fibroblasts (fibr.). OvCar8 and Detroit 551 cells, A2780 and Detroit 551 cells were seeded stained or unstained simultaneously into ULA plate and grown for 96 h. After 96 h of growth, different read outs were performed. (**B**) Representative microscopic images of monocellular and co-cultivated spheroids after 96 h of growth. Scale bar 500 μm. (**C**) Quantitative differences in the size of spheroids after 96 h. Data in floating bar plot (line at mean), one-way ANOVA, ** (*p* < 0.01), *** (*p* < 0.001), **** (*p* < 0.0001). (**D**) Quantitative differences in the compactness of spheroids after 96 h. Data in floating bar plot (line at mean), one-way ANOVA, ** (*p* < 0.01), **** (*p* < 0.0001). (**E-H**) Growth over time. Representative images of OvCar8 (E) and A2780 (G) mono-cultured and co-cultured spheroids with fibroblast. Scale bar 500 μm. Quantification of spheroid size over time of OvCar8 (F) and A780 (H) monocellular and co-cultivated spheroids. Data are means ± SD, *N* = 3.

In this study, a spheroid co-culture model with fibroblasts and ovarian cancer cells was developed. For this purpose, ovarian cancer cells (OvCar8, A2780, UF-403 tumour) were co-seeded with fibroblasts (dermal Detroit 551, peritoneal UF-403 fibroblasts) in ultra-low attachment (ULA) plates and cultured for 96 h (Fig. 2A). For better comparison, spheroids were also cultured in mono-culture consisting of only one cell type. For co-culture, also called multicellular spheroid, the cells were seeded in different ratios of fibroblasts to ovarian cancer cells.

### Simultaneous seeding of cancer cells and fibroblasts

First, co-culture spheroids were generated by simultaneous seeding of cancer cells and fibroblasts. After seeding, the cells of both types mixed passively and formed spheroids of different size and tightness overnight. OvCar8 cells formed stable spheroids after 96 h with significantly increased size compared to mono spheroids when fibroblasts were added in a ratio of 2:1 (Fig. 2B, C). The spheroid area increased from 0.238 mm^2^ to 0.308 mm^2^. A2780 Spheroids formed looser aggregates after 96 h in mono-culture. Interestingly the addition of fibroblasts led to dramatic changes of the spheroids structure, as they became compact and rounded and decreased significantly in size (Fig. 2B, C, D). Addition of 250 fibroblasts to A2780 cells reduced the size from 0.869 mm^2^ to 0.716 mm^2^, and addition of 2,000 fibroblasts resulted in spheroids with a size of 0.376 mm^2^. These spheroids also increased significant in compactness (Fig. 2D; Supplementary Information (Supp Fig. 2)).

Comparing the mono-culture with the co-culture with the highest fibroblast ratio, differences in the course of formation can be observed. OvCar8 co-culture spheroids appeared compact after only 24 h and very round after 48 h (Fig. 2E, F). In mono-culture, the rounding process was delayed by 24 h. During growth, A2780 spheroids showed an even more pronounced change in size (Fig. 2G, H). Moreover, the necrotic core visually enlarged in these co-culture spheroids (Fig. 2G).

**Fig. 3:**
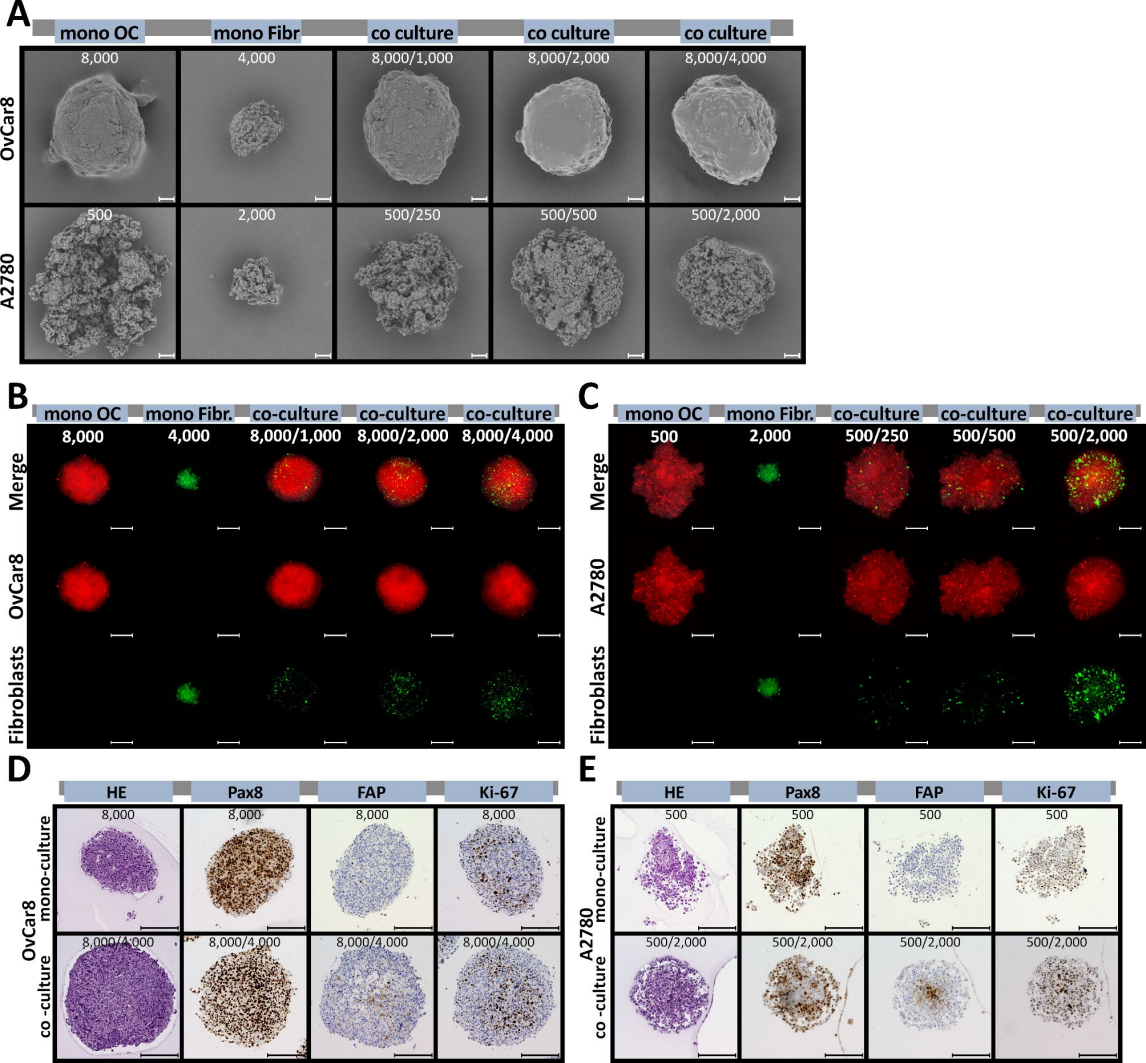
**Microscopic characterisation of the structure of simultaneously seeded mono and co-cultured spheroids.** OvCar8 (OC), A2780 (OC) and Detroit 551 (Fibr.) monocellular and OvCar8 and Detroit 551, A2780 and Detroit 551 multi-cellular spheroids were cultured for 96 h as described in Fig. 2A. (**A**) Spheroids were examined by scanning electron microscopy after 96 h of growth. Scale bar 50 μm. (**B,C**) Prior to cultivation, the cells were stained with fluorescence dyes in order to subsequently assign them to a cell type during cultivation. These spheroids were fixed, cleared and imaged after 96 h of growth using LSM. Red/Cell Tracker Deep Red: ovarian cancer cells; green/CellTracker Green CMFDA: fibroblasts. Scale bar 200 μm. (D, E) Histological and immunohistochemical analysis of spheroid morphology and protein expression of OvCar8 (**D**) and A2780 (**E**) spheroids after 96 h of growth. Ki-67 is a proliferation marker, while PAX8 is marker for ovarian cancer cells and fibroblast activation protein markers fibroblasts, respectively. Scale bar 200 μm.

Furthermore, to depict more details, the spheroids were examined using scanning electron microscopy. As described previously, OvCar8 and A2780 spheroids differed significantly in their phenotype as spheroids^25,27^. However, co-cultivation further altered its morphology (Fig. 3A). OvCar8 showed a smoother surface than in mono-culture. A2780 increased significantly in compactness compared to mono-culture. However, the surface of A2780 spheroids still showed the characteristic juxtaposition of many clusters of lightly packed cells with deep, pore-like structures. In order to better classify the position of the different cell types within the spheroid, the cells were priorly stained with fluorescent dyes. Fluorescent microscopy showed that ovarian cancer cells (red) made up the largest proportion of the spheroid (Fig. 3B, C). However, the fibroblasts (green) were distributed very evenly in the spheroid (show at the bottom of Fig. 3B, C). These findings are also presented in the 2.5D LSM images of the midplane of the 3D spheroids, which can be found in the supplementary information (Supp Fig. 2). In addition to typical HE staining, spheroids were also immunohistological stained with PAX8 as a tumour marker and FAP as a fibroblast marker (Fig. 3D, E). The fibroblasts in the mono-culture were FAP-positive and PAX8-negative. In contrast, it was exactly the opposite in the OvCar8 and A2780 mono-culture. In co-culture, fibroblasts were found to be slightly increased in the core. In A2780 spheroids, this arrangement was even more evident than in OvCar8. The proliferation detected via Ki-67 is clearly present in co-cultures as well as in mono-cultures (Ki-67 Index: A2780 mono-culture: 37 %, co-clture: 38,3 %; OvCa8 mono-culture: 26,0 %, co-clture 21,7 %) (Sup Fig. 1).

**Fig. 4:**
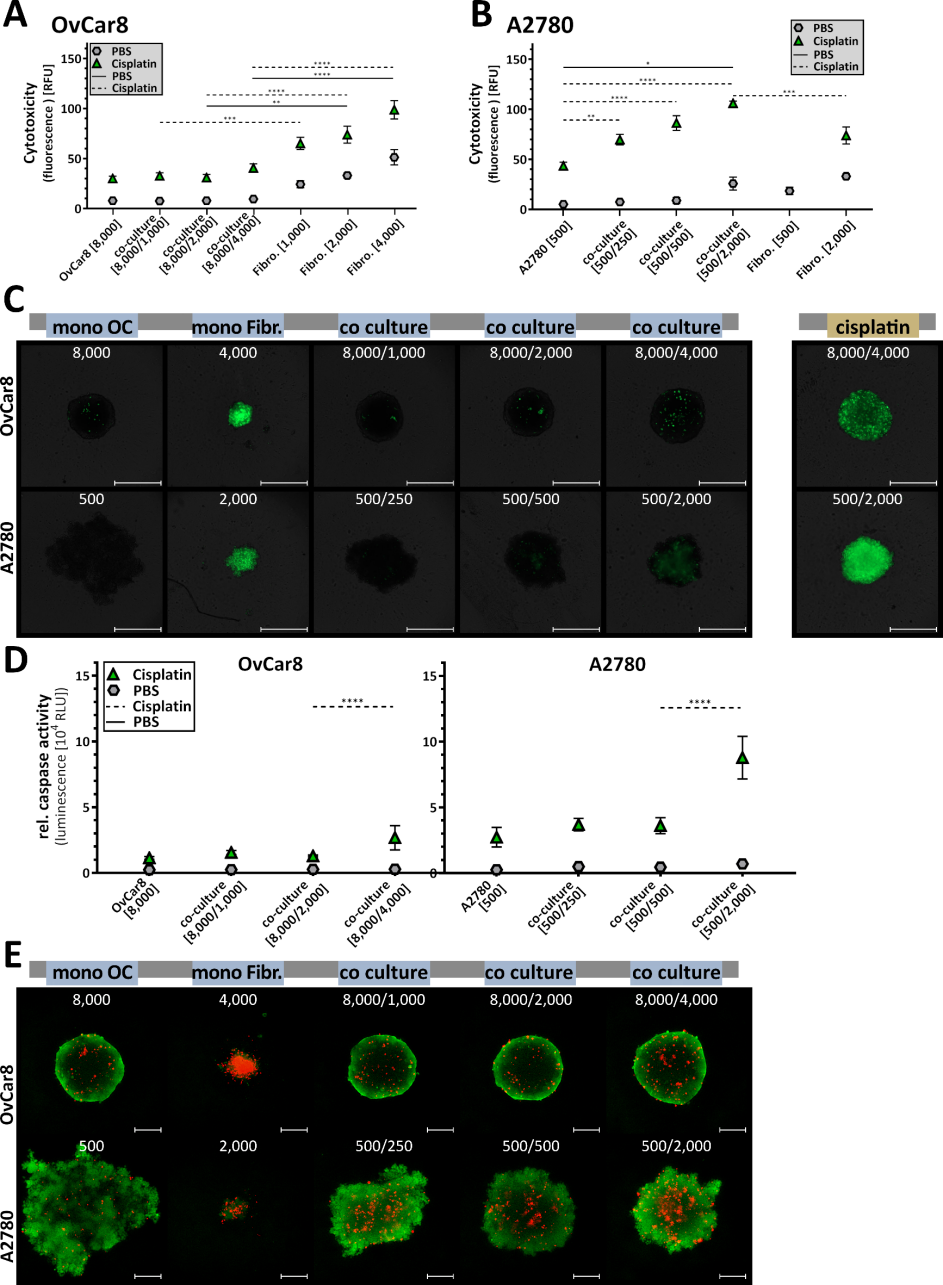
**Cytotoxicity and apoptosis of simultaneously seeded mono and co-cultured ovarian cancer spheroids.** OvCar8 (OC), A2780 (OC) mono-cultured and co-cultured spheroids with fibroblasts (Fibr.) were cultured in ULA plates for 96 h as shown in Fig. 2A. Spheroids were treated after 72 h for 24 h with 100 µM cisplatin or PBS (**A-C**) Cell toxicity was measured by fluorescence microscopy using CellTox Green (timepoint 96 h after seeding). The fluorescence signals after treatment were quantified (relative fluorescence units RFU) (A: OvCar8, B: A2780). Quantitative data are means ± SEM, *N* = 3, one-way ANOVA, * (*p* < 0.05), ** (*p* < 0.01), *** (*p* < 0.001), **** (*p* < 0.0001). Representative images are shown (**C**). Scale bars, 500 μm. (**D**) Following cultivation, viability and caspase activity were measured after 96 h of growth (relative luminescence units RLU). Quantitative data are means ± SEM, *N* = 3, one-way ANOVA, **** (*p* < 0.0001). (E) A further cell viability assay of spheroids was performed using a live/dead staining with propidium iodide and calcein AM. Viable cells appear as green, while nonviable cells appear as red. Scale bars, 200 μm.

As this new model is intended to be used for the study of physiological processes and interactions between tumour and microenvironment, but also for the testing of new drugs, cell toxicity and apoptosis rate of these spheroids after 96 h of growth were investigated. For better analysis, spheroids were treated with 100 µM cisplatin as a positive control in addition to the untreated sample. Our analyses showed increased cytotoxic activity for the untreated (PBS) samples exclusively in the fibroblast mono-culture (Fig. 4A-C). Here, the cytotoxicity increased proportionally to the cell number. Quantitatively, no significant difference in cytotoxicity between co-culture and mono-culture was evident for OvCar8 spheroids (Fig. 4A, C). While the cytotoxicity signal of 7.89 RFU was measured in the mono-culture, the value in the co-culture with 4,000 fibroblasts was 9.49 RFU. Numerically, co-culture was more sensitive to cisplatin treatment than mono-culture. For treatment, the values were 30.11 RFU for the mono-culture and 40.50 RFU for the co-culture with 4,000 fibroblasts.

In contrast A2780 spheroids when co-cultured with highest numbers of fibroblasts show increased cytotoxicity (Fig. 4B, C) with a cytotoxicity signal in mean of 5.12 RFU for mono-culture and 25.77 RFU for co-culture [500/2,000] (one-way ANOVA: mono-culture vs. co-culture [500/2,000] *p* = 0.012). Interestingly, comparing the difference between untreated and treated samples, the co-culture is 2-fold more sensitive to cisplatin than mono-culture. Significant differences between treated mono-culture and treated A2780 co-cultures were detectable and increased with increasing numbers of co-cultured fibroblasts (one-way ANOVA: mono-culture vs. co-culture [500/250] *p* = 0.0045; co-culture [500/500] *p* = 0.0001; co-culture [500/2,000] *p* = 0.0001).

Apoptosis levels also demonstrate this increased sensitivity to cisplatin therapy due to the addition of fibroblasts in spheroid formation (Fig. 4D). Again, of the rate of apoptosis between untreated and treated samples, the OvCar8 co-culture with the highest number of fibroblasts is 2.7-fold more sensitive to cisplatin than the mono-culture, and the A2780 co-culture with the highest number of fibroblasts is 3.2 times more sensitive (one-way ANOVA: mono-culture vs. co-culture [500/2,000] *p* = 0.0001). However, we were not able to infer whether tumour cells or fibroblasts induce increased apoptosis. In addition, live-dead staining was performed to assign signals more clearly (Fig. 4E). The spheroids were predominantly vital.

## Sequential seeding of cancer cells and fibroblasts

**Fig. 5:**
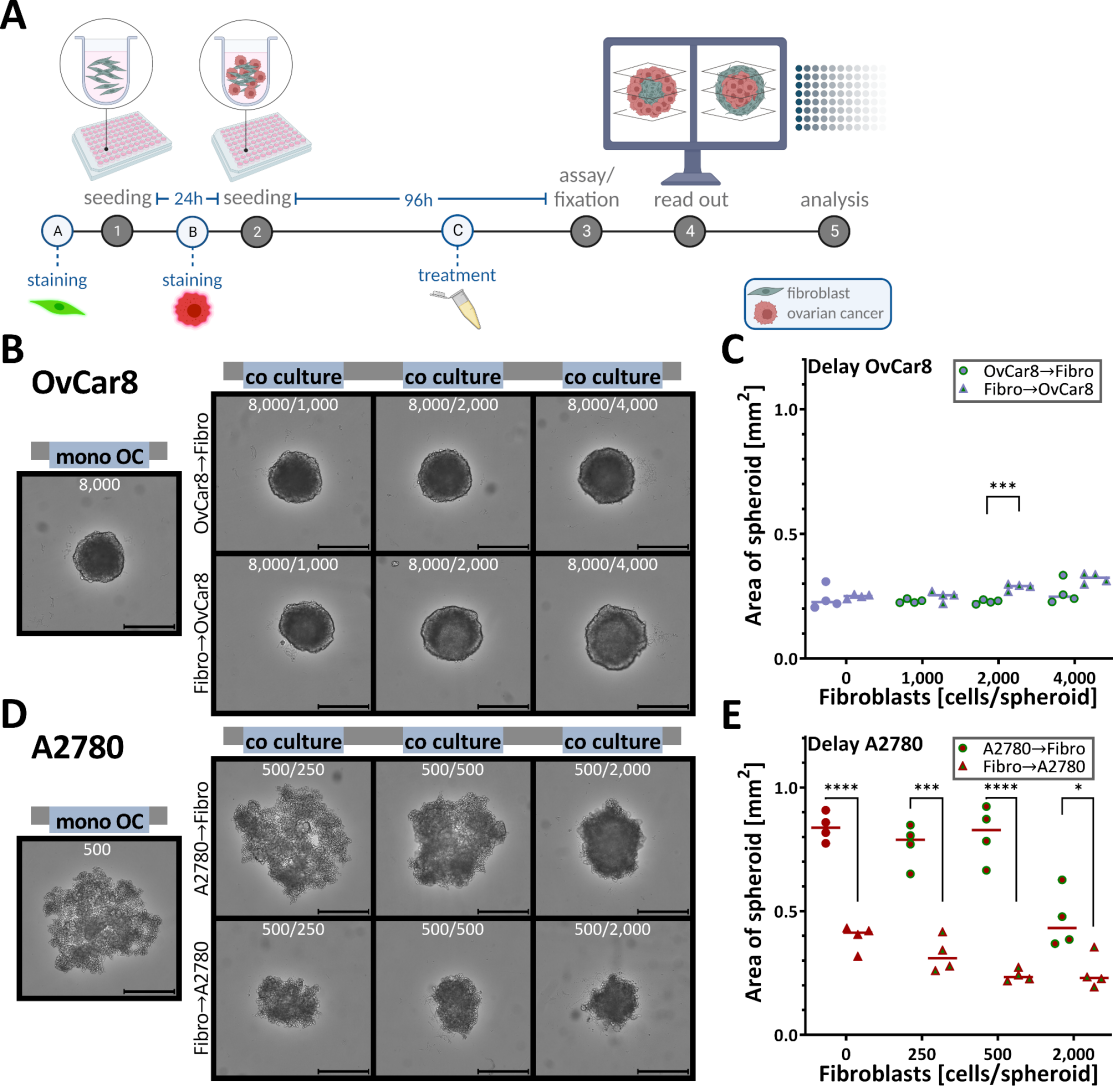
**Growth kinetics of sequentially seeded co-cultured spheroids.** (**A**) Workflow of the newly developed co-cultured spheroids with staggered seeding (with 24 h delay). OvCar8 and Detroit 551 cells, A2780 and Detroit 551 cells were seeded stained or unstained sequential (with 24 h delay) into ULA plates and grown for 96 h (from the time of the first seeding). Ovarian cancer cells were seeded first and then fibroblasts, so also vice versa (first seeded → second seeded). These reverse seeding processes were analysed and compared to each other. (**B**) Representative microscopic images of sequential seeded multi-cellular OvCar8-fibroblast spheroids after 96 h of growth. Scale bar 500 μm. (**C**) Quantitative differences in the size of different OvCar8-fibroblast spheroids after 96 h of growth. Quantitative data, t-test, *** (*p* < 0.001). (**D**) Representative microscopic images of sequential seeded multi-cellular A2780-fibroblast spheroids after 96 h of growth. Scale bar 500 μm .(**E**) Quantitative differences in the size of different A2780-fibroblast spheroids after 96 h. Quantitative data, t-test, * (*p* < 0.05), *** (*p* < 0.001), **** (*p* < 0.0001).

Previous studies describe that fibroblasts often form the core of the tumour *in vivo* and serve as a scaffold for the surrounding tumour cells^12^. Staggered seeding could therefore provide a scaffold to which the other cells can attach. We varied both the time of seeding and the order in which the different cell types were seeded (Fig. 5A). Thus, we could compare simultaneous and sequential (with a delay of 24 h) seeding of different cell types to select the one that better represents the ovarian cancer stromal microenvironment.

For this purpose, the cells were staggered seeded and the growth was observed. 96 h after the first seeding, the spheroids were compared. When fibroblasts were seeded first followed by tumour cells, the size of OvCar8 spheroids slightly increased. For the OvCar8 spheroid with fibroblast scaffold, an enlargement of about 15 % could be reported (Fig. 5B, C). The opposite is true for the A2780 spheroids. The spheroids with tumour cells as scaffolds are significantly larger than the A2780 cells with fibroblasts as scaffolds. In A2780 spheroids scaffolded with fibroblasts, a loss of approximately 40 % was observed (Fig. 5D, E).

**Fig. 6:**
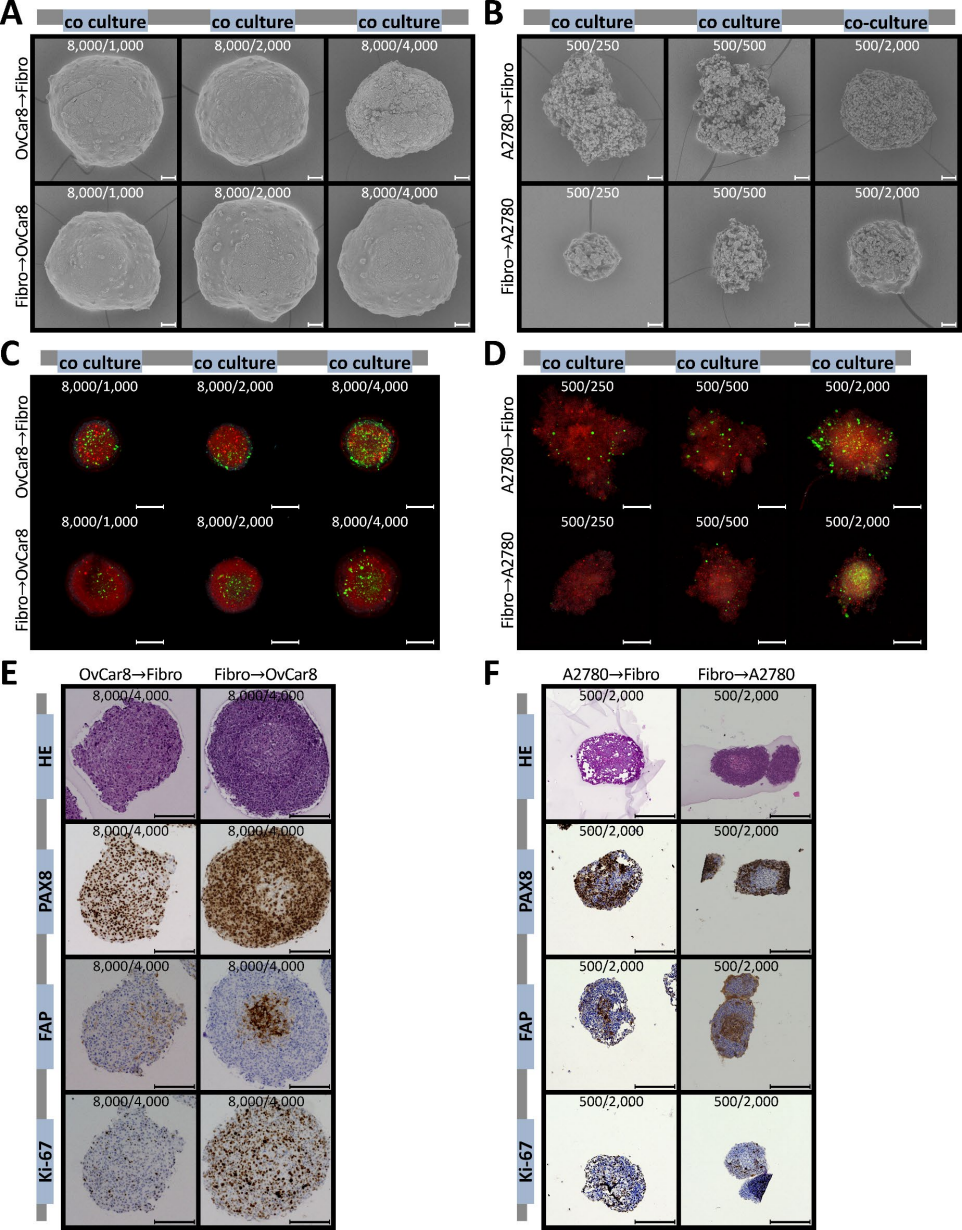
**Morphological characterisation of sequential seeded co-cultured spheroids.** OvCar8 and Detroit 551, A2780 and Detroit 551 co-cultured spheroids were seeded time-shifted and cultured for 96 h as described in Fig. 2A. (**A,B**) Spheroids were examined by scanning electron microscopy after 96 h of growth. Scale bar 50 μm. (**C,D**) Prior to cultivation, the cells were stained with different fluorescence dyes in order to subsequently assign them to a cell type during cultivation. These spheroids were fixed, cleared and imaged after 96 h of growth using LSM. Red/Cell Tracker Deep Red: ovarian cancer cells; green/CellTracker Green CMFDA: fibroblasts. Scale bar 200 μm. (**E,F**) Histological and immunohistochemical analysis of spheroid morphology and protein expression of spheroids after 96 h of growth. Ki-67 is a proliferation marker, while PAX8 is marker for tumour cells and fibroblast activation protein markers fibroblasts, respectively. Scale bar 200 μm.

Scanning electron micrographs were taken to further characterize the surface structure. No changes were detected in OvCar8 spheroids between simultaneous and both delayed seedings (Fig. 6A). In contrast, A2780 spheroids had more ruffled surface with merged structures, more integrity and fewer clusters in the spheroids with fibroblast scaffolds (Fig. 6B). To determine the position of fibroblasts and tumour cells in the spheroid, fluorescence staining was used. Figure 6C, D shows that when the tumour cells were seeded second, they attached to the fibroblast scaffold and vice versa. The fluorescent signal from the core decreased because spheroids are not completely transparent. This complicated the analysis of the interior by fluorescence microscopy. Fluorescence analysis indicated mild invasion of the fibroblast scaffold by tumour cells (Fig. 6C, D). Immunohistochemistry confirmed this minor invasion (Fig. 6E, F). Interestingly, proliferation was significantly increased in the spheroids with fibroblast scaffold (Ki-67 Index: A2780→fibroblasts: 12,1 %, fibroblasts→A2780: 40,4 %; OvCar8→fibroblasts: 12,1 %, fibroblasts →OvCar8: 68,1 %) (Supp Fig. 1). If tumour cells were first seeded, there was no defined area of only tumour cells or only fibroblasts, but an equal mixture of both cell types throughout the spheroid.

**Fig. 7:**
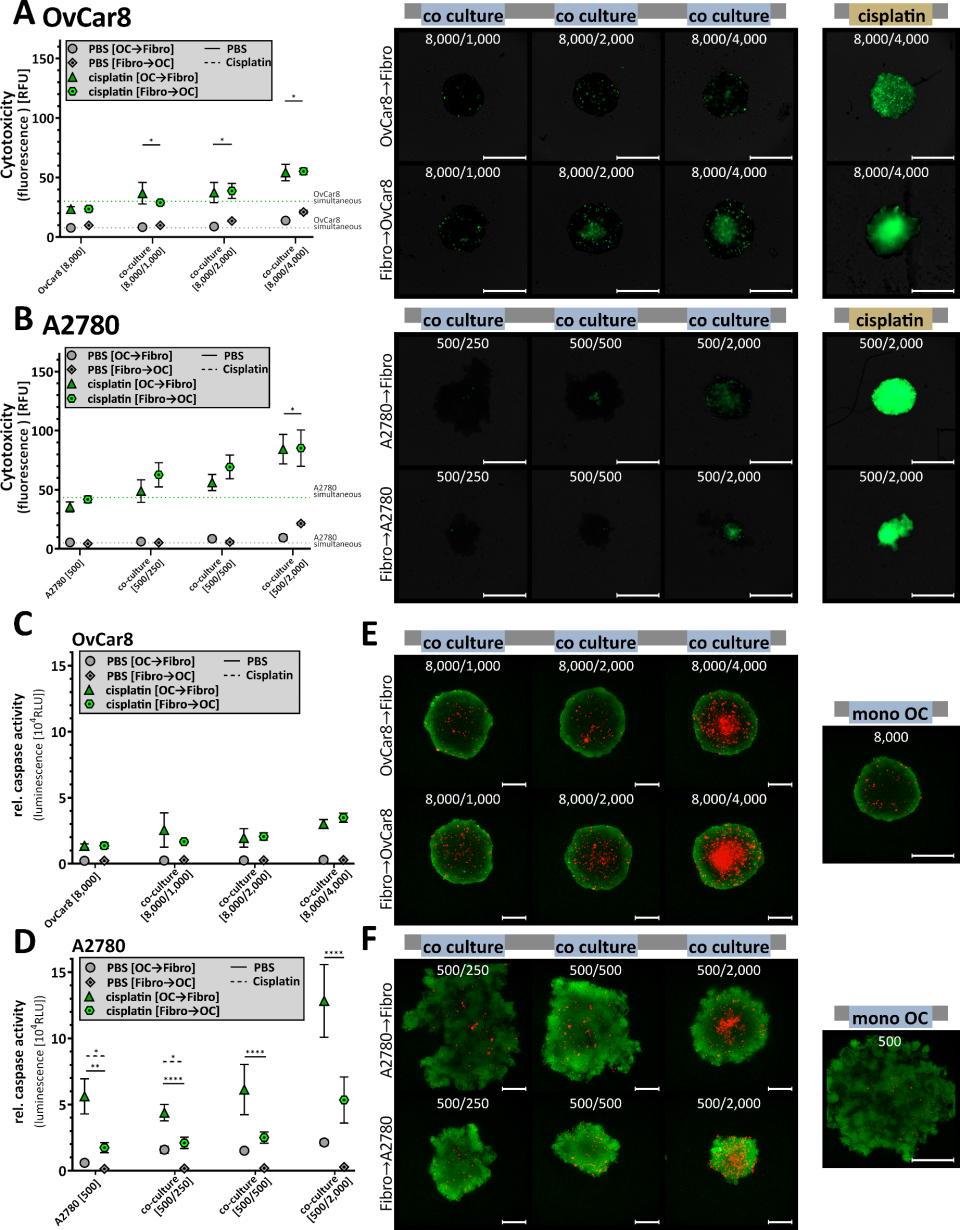
**Cytotoxicity and apoptosis of sequential seeded co-cultured ovarian cancer spheroids.** OvCar8 and Detroit 551, A2780 and Detroit 551 co-cultured spheroids were seeded time-shifted and cultured for 96 h as described in Fig. 2A. Spheroids were treated after 72 h for 24 h with 100 µM cisplatin or PBS. (**A,B**) Cell toxicity of OvCar8-fibroblast (**A**) and A2780-fibroblasts (**B**) spheroids was measured by fluorescence microscopy using CellTox Green 24 h after treatment. Scale bars, 500 μm. The fluorescence signals after treatment were quantified (relative fluorescence units RFU). Quantitative data are means ± SEM, *N* = 3, t-test * (*p* < 0.05). (**C,D**) Viability and caspase activity were measured after 24 h treatmet (**C**: OvCar8,** D**: A2780) (relative luminescence units RLU). Quantitative data are means ± SEM, *N* = 3, t-test * (*p* < 0.05), ** (*p* < 0.01), **** (*p* < 0.0001). (**E,F**) Viable cells were stained with calcein-AM in green, while death cells appear in red by propidium iodide (E: OvCar8, F: A2780). Scale bars, 200 μm.

Comparing the cytotoxicity of the different seedings, the OvCar8 spheroids showed a slightly increased toxicity for the spheroids with fibroblast core, which in turn increased with increasing fibroblast number (Fig. 7A). Significant differences between untreated sequential seeded OvCar8 co-culture were measured (t-test: co-culture [8,000/4,000] *p* = 0.0169). Sensitivity to cisplatin increased with increasing numbers of fibroblasts. However, an increase due to the order of seeding was not detectable. Untreated A2780 spheroid co-cultures induced similar cytotoxicity as mono-cultures, except for the spheroids with fibroblast core and high fibroblast number (Fig. 7B) (t-test: co-culture [500/2,000] *p* = 0.0148). Sensitivity to cisplatin increases with increasing fibroblast number. However, there was no difference in the order of seeding.

Apoptosis induction in OvCar8 co-cultures did not differ by seeding method (Fig. 7C). In contrast, A2780 spheroids exhibited significantly reduced apoptosis rates in spheroids scaffolded with fibroblasts (Fig. 7D) (t-test untreated co-culture [500/250], [500/500] and [500/2,000] *p* < 0.0001). However, the spheroids with tumour scaffolds were more sensitive to cisplatin than those with fibroblast scaffolds. Figure 7E and F demonstrate the ratio of live to dead cells in the spheroid. These results were consistent with the cytotoxicity results in Fig. 7A, B. OvCar8 spheroid co-cultures had an increased percentage of dead cells, which increases with fibroblast scaffolding. The A2780 spheroids showed rarely change in the number of dead cells due to different seeding methods. Fibroblasts were present in increased numbers dead in the core.

## Simultaneous seeding of primary ovarian tumour cells and fibroblasts of the same patient

Primary ovarian cancer cultures are a powerful tool for studying cancer biology and physiology. The use of primary ovarian cancer cells provides a more accurate picture of tumours and is more suitable for preclinical analyses, in contrast to the most commonly used immortalized cell lines, which may not be fully informative about cancer properties. In addition, primary cultures can reflect the natural microenvironment of a tumour and provide information about behavioural patterns during cancer development, progression, and metastasis.

For this reason, we not only used established cell lines but also with primary ovarian tumour cells and primary fibroblasts from the same patient to develop a 3D co-culture. The tumour cells were derived from a surgery in which healthy peritoneum tissue was also removed. After this sample was cultivated, it was used for 3D co-culture.

**Fig. 8:**
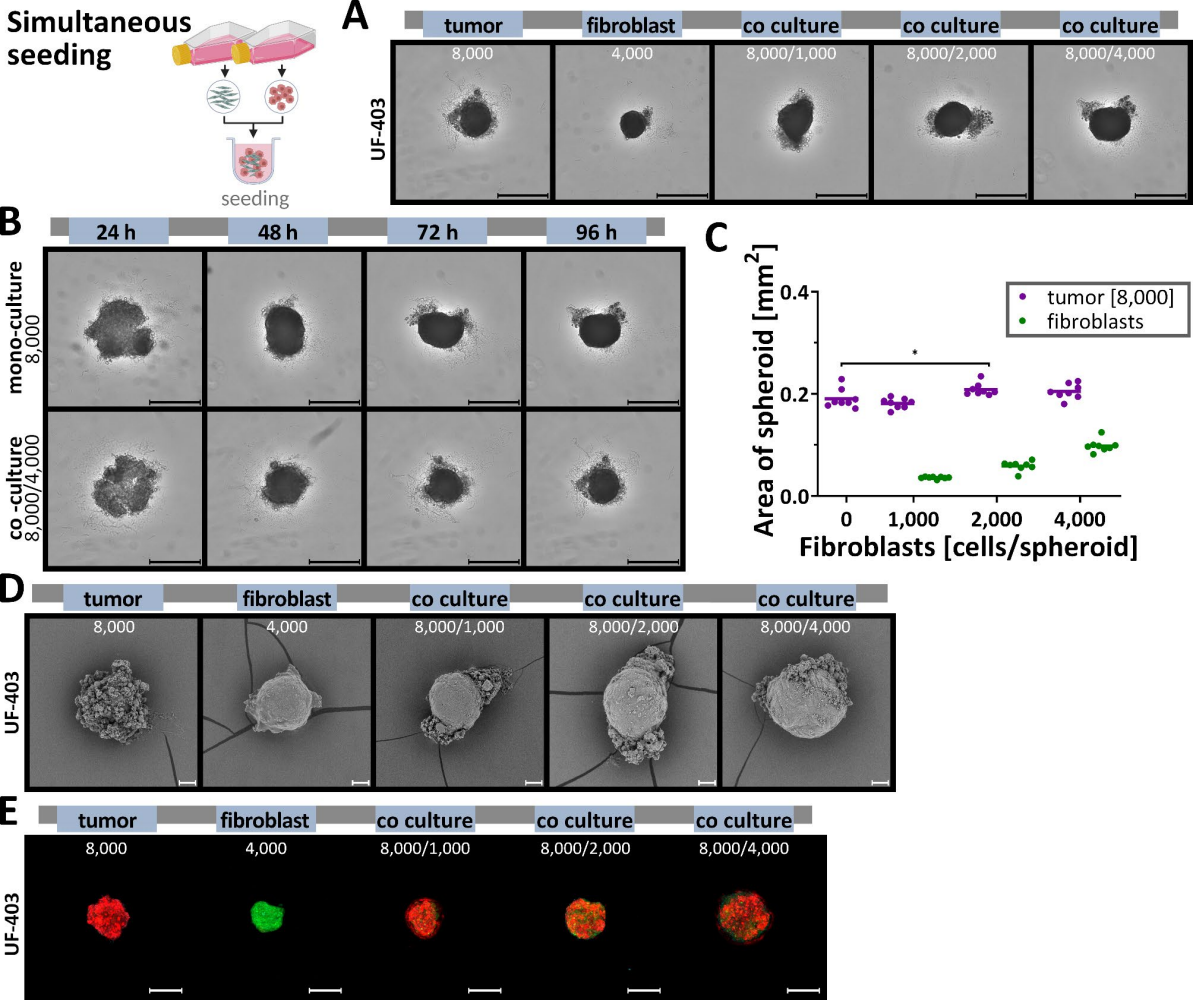
**Characterisation of growth and morphology of simultaneously seeded mono and co-cultured spheroids from primary patient cells.** Primary ovarian cancer cells and human peritoneal fibroblasts of the same patent were seeded stained or unstained simultaneously into ULA plate and grown for 96 h. Spheroids were cultured in mono-culture and in co-culture with ovarian cancer cells and different cell numbers of fibroblasts. After growth, various assays were performed. (**A**) Representative microscopic images of mono-cultured and co-cultured spheroids after 96 h of growth. Scale bar 500 μm. (**B**) Representative microscopic images of mono-cultured and co-cultured spheroids at 24-hour intervals. Scale bar 500 μm. (**C**) Quantitative differences in the size of spheroids after 96 h of growth, one-way ANOVA, * (*p* < 0.05). (**D**) Spheroid surface were analysed by scanning electron microscopy after 96 h of growth. Scale bar 50 μm. (**E**) Prior to cultivation, the cells were stained with different fluorescence dyes in order to subsequently assign them to a cell type during cultivation. These spheroids were then imaged after 96 h using LSM. Red/Cell Tracker Deep Red: ovarian cancer cells; green/CellTracker Green CMFDA: fibroblasts. Scale bar 200 μm.

Tumour cells and fibroblasts formed stable spheroids as mono-culture and co-culture during 96 h of growth, with only minor differences in size due to co-culturing (Fig. 8A, B, C). Here, two different cell numbers of primary ovarian tumour cells were seeded and co-cultured with fibroblasts. However, differences in surface structure between fibroblasts and tumour spheroids were obvious (Fig. 8D). Mono-cultured fibroblasts were highly cohesive, showing tight intercellular connections forming a flat surface similar to OvCar8 spheroids (Fig. 8D, 2nd Fig. from left). In contrast, mono-cultured tumour spheroids revealed reduced cortical tension and conglomerated cell–cell adhesion (Fig. 8D, 1st Fig. from left).

**Fig. 9:**
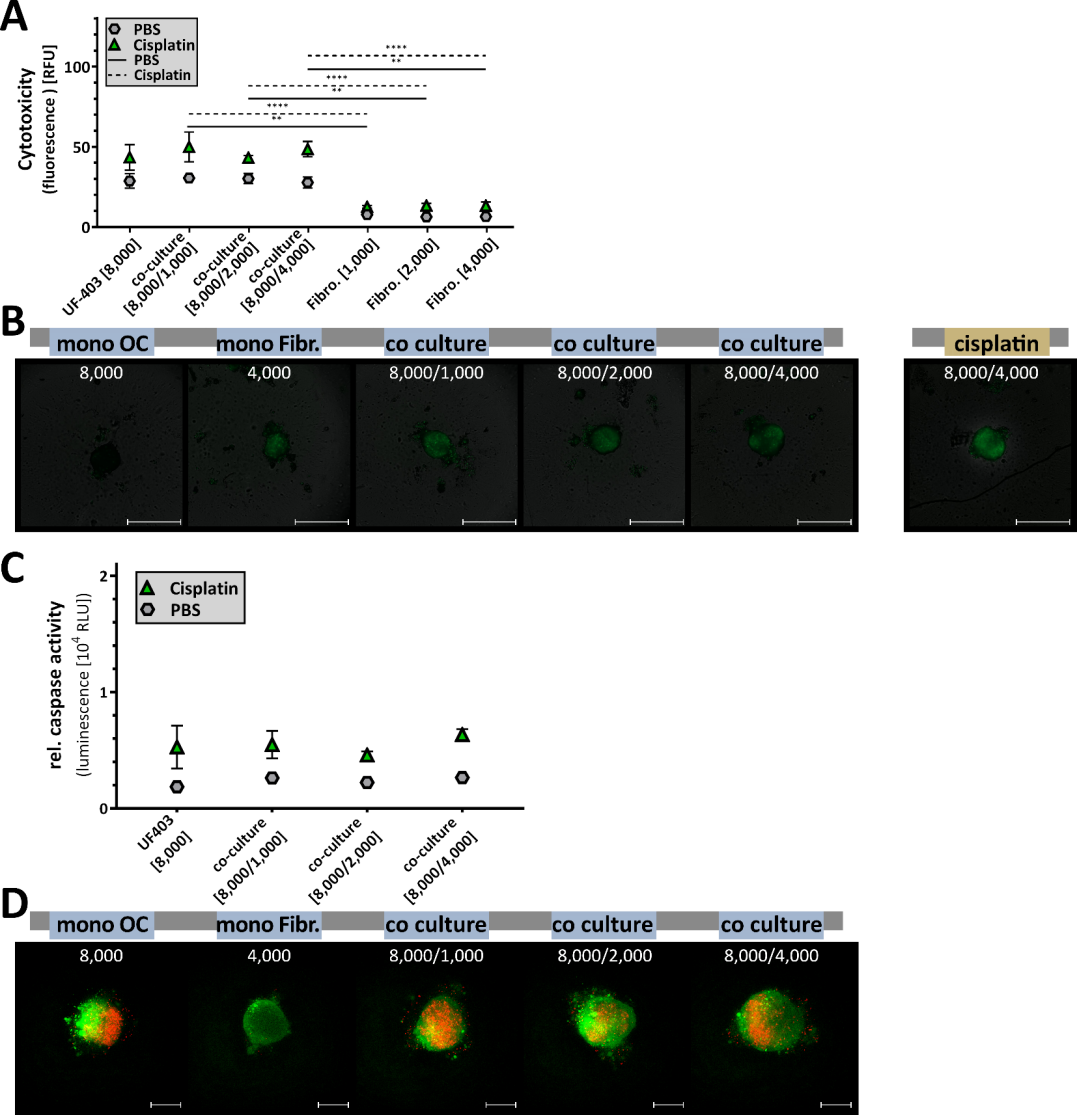
**Cytotoxicity and apoptosis of simultaneously seeded mono and co-cultured spheroids from primary patient cells.** Primary ovarian cancer cells and human peritoneal fibroblasts of the same patient were seeded into ULA plate and grown for 96 h as described above. Spheroids were treated after 72 h for another 24 h with 100 µM cisplatin or PBS. (A, B) Cell toxicity of UF-403-co-culture spheroids was measured by fluorescence microscopy using CellTox Green 24 h after treatment. (**A**) The fluorescence signals after treatment were quantified (relative fluorescence units RFU). Quantitative data are means ± SEM, *N* = 3, one-way ANOVA, ** (*p* < 0.01), **** (*p* < 0.0001). (**B**) Representative microscopic images of monocellular and multicellular spheroids after CellTox Green staining. Scale bar 500 μm. (**C**) Viability and caspase activity were measured after 24 h treatment (relative luminescence units RLU). Quantitative data are means ± SEM, *N* = 3, one-way ANOVA. (**D**) Viable cells were stained with calcein-AM in green, while death cells were marked in red with propidium iodide. Scale bars, 200 μm.

Primary co-culture spheroids contained two distinct cell layers; the inner layers were densely packed, while the outer layers interacted much more loosely (Fig. 8D). It appeared that the closely cooperating fibroblasts formed the inner core, while the tumour cells adhered to the outside, confirmed by fluorescence staining of the cells. The green fibroblasts form the scaffold for the red-fluorescent tumour cells (Fig. 8E). The cytotoxicity and viability of the cells were comparable to mono-cultures. However, the primary cells were less sensitive to cisplatin than the cell lines. Here, the co-cultivation was equivalent to the mono-cultivation (Fig. 9A-C). Also, triple staining with propidium iodide, calcein-AM and Hoechst 33342 did not reveal increased general toxicity without treatment due to co-cultivation of primary cells (Fig. 9D).

## Discussion

The aim of the present study was to develop a spheroid model more closely to the *in vivo* environment of ovarian cancer compared to monocellular 3D tumour models. In this context not only the growth of tumours but also the changes due to interaction with the microenvironment is of major interest.

In ovarian cancer metastasis, malignant cells detach from the primary ovarian tumour and colonise in the abdominal cavity as multicellular aggregates called spheroids, leading to secondary metastasis^33^. Fibroblasts, which are part of the microenvironment, not only play an important role in tumourigenesis and proliferation, but are also significantly involved in the formation of these heterotypic spheroids^34–36^. CAF have the potency to form the core of these spheroids and may serve as scaffolds for attachment of floating tumour cells^18,37^.

It is already proven that spheroid formation can influence the efficiency of tumour therapy significantly^38,39^. Therefore, it is important to develop test models considering biological processes in tumour progression. Since the 1990s, there have been ovarian cancer models using spheroids as a 3D system for testing new drugs^40^. However, the influence of fibroblasts as scaffold formers and interaction partners have not been extensively considered so far. The development of a suitable 3D tumour model that also represents the TME is not completely new. There are different models that also culture ovarian cancer cells with TME components. For example, Baka et al. produces a 3D bioprinting model from OC and fibroblasts^41^. Shishido et al. provided an *in vitro* 3D spheroid model of ovarian cancer cells with rat mesothelial cells^42^. Chip systems are also used in this context, as shown by Ibrahim et al.^43^. Malacrida et al. try to reconstruct a 3D tissue by using a tetra-culture model comprising human primary omental adipocytes, fibroblasts, mesothelial cells and early passage HGSOC^44^. However, these models are limited both by their complex implementation and by the indirect 2D cultivation of the TME components. For this reason, we have developed a new model in which fibroblasts and tumour cells are cultured simultaneously. To increase the translational aspect, we employed primary *ex vivo* tumour cells and fibroblasts from the same patient.

Our work showed that co-culture with fibroblasts induced tumour cells to form more solid 3D structures that with differ in size. The differences were more pronounced in loose aggregate-forming tumour cells such as A2780 than in spheroid-capable tumour cells. Moreover, the formation of solid spheroids accelerated over time. When both cell types are seeded simultaneously, fibroblasts were found in higher numbers in the core of the spheroid and forming the scaffold, very similar to the situation *in vivo*^34,35^. This phenomenon was even more evident in the sequential seeding with fibroblasts seeded first. In reverse seeding, fibroblasts slowly infiltrated tumour cells instead of encasing the tumour cells. During spheroid formation, loose cell aggregates form through integrin-ECM binding and subsequently compact spheroids form through haemophilic cadherin-cadherin interactions^45^. Interestingly, the more fibroblasts formed the scaffold of the tumour, the more proliferation increased. Thus, the order of seeding has a decisive influence on the architectural arrangement of cells in the spheroid, accompanied by the influence on proliferation. It is already known that CAFs promote growth, proliferation and metastasis via mediators such as COL 1A1, FGF-1 or HGF. Among others, the extracellular-signal regulated kinase (ERK) signalling pathway is activated^46–48^.

Assuming that co-cultivation also has a significant effect on morphology, this became clearly evident in the changes of the spheroids´ size. However, surface structure of the spheroids determined via scanning electronic microscopy showed remarkable differences. The A2780 spheroids form a surface structure similar to that of the mono-culture when seeded simultaneously and sequentially with tumour cells as scaffolds. However, when fibroblasts formed the scaffold, sequential seeding led to a much smoother surface with a merged aspect.

To establish a model for new drug testing, it is critical that the system itself does not exhibit high cell death rate without the influence of a drug. Otherwise, effect sizes of agents are more difficult to measure and assessments or predictions are less transferable to *in vivo*. For this reason, the cytotoxicity and apoptosis of the co-cultures were studied with and without drugs. It is well known that spheroids consist of three layers. The proliferative layer is the outermost layer and is metabolically active. The middle layer is the quiescent layer, while in the core there are necrotic cells due to nutrients and oxygenation deficiency^49,50^. More compactness results in a larger apoptotic/necrotic core as they have poorer access to nutrients and oxygen and consequently promote the formation of a hypoxic central region^51,52^. Overall, the results indicated good applicability of our model. However, it can be concluded that the ratio of cell types should always have more tumour cells than fibroblasts, in our hands the ratio 2:1 (OvCar8 or UF-403 : fibroblasts) and 1:1 (A2780 : fibroblasts). Interestingly, the co-cultures, especially with dermal fibroblasts, were more sensitive to cisplatin than mono culture. In primary cultures, tested patient cells were generally more resistant to cisplatin than the cell lines. New evidence shows that cancer-associated fibroblasts can promote or inhibit drug sensitivity. Remsing et al. found that mutation of the KRAS protein in co-cultures leads to resistance to MEK inhibitors. Cancer-associated fibroblasts sensitized tumour cells with a mutated EGFR protein to EGFR inhibitors^53^. Morales et al. indicate that normal fibroblast supernatants potentialize the therapy’s efficiency, whereas cancer-associated fibroblast secretomes favour melanoma cell survival^54,55^. Peltanova et al. shows that CAFs can promote and/or inhibit colony-forming ability and cisplatin resistance of head and neck squamous cell carcinoma cells^56^. Also an increased development of resistance by fibroblasts is described^35,57^. CAFs can activate the Wnt/β-catenin signalling pathway in OC cells and induce epithelial-mesenchymal transition (EMT) and cisplatin resistance^58^. Cysteine and glutathione, which are produced by CAFs, are also thought to inhibit the accumulation of cisplatin in OC tumour cells, leading to platinum resistance^59^. When interpreting our results, it must also be observed that fibroblasts promote the growth of cancer cells and can therefore lead to increased cytotoxicity signal. Nevertheless, this study shows that cisplatin is still effective. Furthermore, cisplatin resistance is not analysed in the present model. Resistance develops in the course of therapy. In order to analyse this, further long-term treatment studies need to be carried out.

If model conditions were prioritized, simultaneous administration of both cell types have major advantages. On the one hand it is the simplest handling. On the other hand, this model conditions show great similarities to the *in vivo* situation of spheroids described in the literature^12,34–36^. Thus, communication and interaction of both cell types occur directly at the formation of the spheroids. In this condition, fibroblasts form the scaffold to which the tumour cells can attach well. However, this requires the identification of the seeding the correct ratio of tumour cells to fibroblasts. For A2780, this ratio is 1:1, while for OvCar8 and UF-403 it is 2:1 (tumour : fibroblasts). Last but not least, the selected conditions, simultaneous seeding at optimum ratio, exhibit low self-cell death rate. The selection of different seeded cell numbers of each cell line is due to the different growth. Also, the selection of the fibroblast number is due to the different growth of the tumour cells.

In addition to the major advantages of this study, a limiting factor is the use of normal skin fibroblasts instead of CAFs in co-culture with cell lines. The Detroit 551 fibroblasts used were originally normal human embryonic skin fibroblasts from a female patient^60^. These cells exhibit high FAP expression, which is a biomarker for CAFs^41,61^. However, this could simply be due to cell immortalization rather than CAF function, as high FAP expression of Detroit551 was present even in the absence of cancer cells. While it is known that normal fibroblasts can transform into CAFs and exert a major influence on tumour cells as CAFs^20^. However, since CAFs can only be acquired as primary cells and occur in very different subtypes, we decided to use normal fibroblasts in co-cultivation with cell lines. To also investigate the influence of tumour proximal fibroblasts on co-culture, the model was repeated with primary fibroblasts and tumour cells.

Weydert et al. developed a fully cost-effective 3D heterotypic model consisting of a human ovarian cancer and a mouse fibroblast cell line^62^. Tofani et al. designed a 3D co-culture model using SKOV-3 and mesenchymal cells or fibroblasts^63^. It is also described that tumour-associated macrophage indicate spheroid formation and valide, novel ovarian cancer 3D immune oncology co-culture model were designed^64,65^. Co-culturing with fibroblasts has been performed in studies with colorectal cancer or breast cancer showing very similar results^66–68^. Different methods have been used, such as the hanging drop method, the liquid overlay technique, and the Collagen Matrix-Incorporated Microfluidic Chip technique.

In the future, this model can be used to further characterize physiological processes and interactions as well as the role of fibroblasts in tumorigenesis, in addition to drug testing. It would be important and interesting to further investigate the protein profile of the co-cultured cells in more detail to further elucidate tumour biological processes and to find potential targets for inhibiting the interaction between tumour and stroma. Furthermore, this model can be used to investigate whether the presence of a complex microenvironment can alter the sensitivity of cancer cells to chemotherapy and whether the treatment resistance observed *in vivo* can be better mimicked and analysed using this model.

The developed model allows evaluation of the interaction in the complex network of tumour and TME and can be a useful tool for further study of biological processes and drug testing. To form an all-encompassing model, the combination with other microenvironment components is essential. However, to understand which component has which influence, it is important to consider the factors individually with the tumour. Thus, this model is an evolving model that is planned to include other components of the microenvironment such as the stroma in the future.

## Conclusions

In summary, we have succeeded in developing and characterizing a more realistic and informative 3D co-culture spheroid model that will improve the testing of new drugs and physiological processes. This involved co-cultivation of tumour cells with fibroblasts. The interaction forms a tumour-like microenvironment that promotes matrix production and proliferation. This study was used to characterize the influence of fibroblasts on tumour cells before further planned studies investigate the influence of other TME components on tumour behaviour.

The interaction of fibroblasts and tumour cells resulted in more stable spheroids, which hardly differed in their surface structure from the mono-culture. Based on optimized tumour cell concentrations in mono-culture, we adapted different co-cultivation modalities of fibroblasts. Simultaneous seeding ensures a direct interaction of cell types and thus a homogeneous distribution more in line with the clinical situation.

All in all, this study provides a basis to incorporate the tumour environment into the consideration of tumour behaviour and response to therapeutics. It is thus fundamental work for the further development of *in vivo* mimicking models.

## Materials and methods

### Cell culture conditions

The human ovarian cancer cell lines OvCar8 and A2780, and human fibroblasts Detroit 551 were purchased from American Type Culture Collection (ATCC). Cell lines were maintained in DMEM high glucose medium, supplemented with 10 % fetal bovine serum (FBS), 60 IU(µg)/mL penicillin–streptomycin (P/S).

Primary cells were isolated from advanced stage ovarian cancer patients during surgery at first diagnosis (UKSH, Campus Kiel). Primary ovarian tumour cells and primary fibroblasts were originated from the same patient (UF-403). The primary ovarian tumour cells were extracted from tumour tissue as described previously^69^. Primary ovarian cancer cells were isolated with RPMI containing 10 % FBS and 60 IU(µg)/mL P/S and transferred to primary fibroblast medium (DMEM high glucose supplemented with 20 % FBS, 60 IU(µg)/mL P/S, Insulin-Transferrin-Selen (ITS) solution I (0.1x) and 0.1 µM dexamethasone) after thawing. Primary fibroblasts were extracted from the paracolic gutter and isolated in a similar process to tumour cells. However, the growth medium for primary fibroblasts consisted of DMEM high glucose supplemented with 20 % FBS, 60 IU(µg)/mL P/S, ITS I (0.1x) and 0.1 µM dexamethasone.

Cells were grown at 37 °C, 5 % CO_2_ in a humidified incubator. Cell authenticity was checked by short tandem repeat (STR) profiling (16) and mycoplasma contamination was routinely investigated, using MycoAlert (Lonza, Basel, CH).

To further characterize malignancy, the polyploid character of tumour cells and fibroblasts were checked, using the TERC (3q26) / MYC (8q24) / SE 7 TC (Kreatech/Leica, #KBI-10704) tricolor probe for fluorescence in situ hybridization (FISH), as described previously^70^. In addition, tumour cells and fibroblasts were stained with the anti-fibroblast antibody 1:50 conjugated to Phycoerythrin (#130-126-007, Miltenyi Biotec, Bergisch Gladbach, DE).

Informed consent was obtained from all donors, in agreement with the approval from the Institutional Ethical Review Board of the UKSH, Campus Kiel (AZ: D578/20).

### Generation and anaylsis of spheroids

The cells were initially seeded in 96-well, ultra-low attachment plates (ULA) (Corning 4520, Corning 7007 or BIOFLOAT Corning, New York, Facellitate Mannheim, DE) centrifugated (3 min, 300x*g*) and maintained for 96 h. Cells were mono-cultured or co-cultured at different ratios of cancer cells and fibroblasts:

**Table:** 

Cells/well		Fibroblasts (Detroit 551)			Spheroid medium
OvCar8	8,000	4,000	2,000	1,000	DMEM medium, 10 % FBS, 60 IU(µg)/mL P/S
A2780	500	2,000	500	250	
		Fibroblasts (UF-403)			
UF-403 Tumour	8,000	4,000	2,000	1,000	DMEM medium, 20 % FBS, 60 IU(µg)/mL P/S, ITS I (0.1x), 0.1 µM dexamethasone

In this work, we also compared simultaneous and sequential (with 24-hour delay) seeding of different cell types. Spheroid formation was monitored daily using the automated cell imager NYONE Scientific (SYNENTEC, Elmshorn, DE) with 4× magnification objective and the settings: brightfield: Ex: BF; Em: Green (530/43 nm). The NYONE Scientific (SYNENTEC, Elmshorn, DE) *spheroid quantification* processor was used to determine the spheroid size and growth kinetics.

### Scanning Electron Microscopy

The spheroids were cultured as described in section Generation and anaylsis of spheroids. For scanning electron microscopy analysis, spheroids were fixed with 2.5 % glutaraldehyde for 1 h at room temperature. After washing, the second fixation was performed using 1 % osmium tetroxide for 1.5 h at room temperature. The spheroids were washed and fully dehydrated in a graded series of ethanol solutions [25 %, 50 %, 75 %, 96 %, 100 %] and air dried on aluminium stubs with carbon adhesive overnight using hexamethyldisilazane. To guarantee sample grounding and to minimise charging effects, fixed samples were gold-sputtered with SCP 050 Sputter Coater (Bal-Tec, Balzers, LI). All images were taken at 500× or 2,500× magnitude with the use of a backscatter detector (Phantom XL, Thermo Fisher, Darmstadt, DE).

### Fluorescence imaging (LSM)

To determine the arrangement of the cell types in the spheroid, we labelled the different cell types with distinct fluorescent dyes before co-culture. Prior to seeding, 10^6^ cell/ml ovarian cancer cells were stained with Cell Tracker Deep Red (Invitrogen #C34565, Willow Creek, Eugene) using serum-free media (A2780: 5 µM, OvCar8: 2 µM, UF-403: 1 µM). After incubation for 1 h at 37 °C, cells were centrifuged again (3 min 300x*g*) and resuspended with fresh medium. Fibroblasts were labelled with CellTracker Green CMFDA (Invitrogen, Willow Creek, Eugene) according to the manufacturer’s protocol before seeding. After staining, the cells were counted using CellDrop Cell Counter (DeNovix, Wilmington, DE) and seeded as described above. After 96 h of growth in ULA plates, spheroids were fixed with 4 % PFA (1 h at 37 °C), transferred into PCR-tubes, followed by washing with PBS supplemented with 1 % FBS. Then, the spheroids were incubated with 0.5 M glycine in PBS for 1 h at 37 °C and with a penetration buffer (0.2 % Triton X-100, 0.3 M glycine and 20 % DMSO in PBS) for 30 min room temperature. The nuclei were stained using Hoechst 33,342 (1:5000) for 3 h at 37 °C. The spheroids were washed in a washing buffer (0.2 % Tween 20, 10 µg/mL heparin, 1 % BSA in PBS) overnight. The spheroids were transferred to glass-bottomed chamber slides (Ibidi # 81817, Gräfelfing, DE) and wetted with 88 % glycerol as a clearing procedure shortly before measurement. Fluorescence imaging was performed using the Zeiss LSM 880 microscope (Carl Zeiss Microscopy). For evaluation, the ZEN 3.5 (blue edition) (Carl Zeiss Microscopy, Oberkochen, DE) was used.

### Immunhistochemical analysis

The spheroids were cultured as described in section generation and anaylsis of spheroids Supernatant was removed by gentle aspiration and spheroids were transferred to 0.5 µl tubes. Subsequently, the spheroids were taken up in 3 % warm agarose to transfer them to parafilm. To locate these agarose particles in paraffin, the agarose was then stained externally with haematoxylin. The solidified agarose was placed in histology cassettes for subsequent fixation in 4 % formaldehyde and dehydration with an ethanol series before embedding in paraffin.

Spheroid sections were cut using a microtome; the tissue sections were deparaffinized and rehydrated in water.

For immunohistochemistry, sections were stained with antibodies directed against Ki-67 (dilution 1:100, clone SP6, ThermoFisher Scientific), PAX8 (dilution 1:50, clone MRQ-50, Cell Marque), FAP Fibroblast activation protein (dilution 1:400, clone EPR20021, Abcam). Antigen retrieval was achieved with ER2 (EDTA-buffer Bond pH 9.0; 20 min for Ki-67; 30 min for PAX8) or with ER1 (citrate buffer Bond pH 6.0; 20 min for FAP). The immunoreaction was visualized with the Bond Polymer Refine Detection Kit (DS 9800; brown labelling; Novocastra; Leica Biosystems GmbH, Nussloch, DE) resulting in a brown colour and counterstained with haematoxylin. The IHC stainings were carried out on the autostainer BOND RX system (Leica Biosystems GmbH, Nussloch, DE). The stained tissue sections were imaged and analysed under the BZ-X800 (Keyence, Osaka, JPN) microscope at 10x magnification objective.

### Viability, apoptosis and Cytotoxicity

Cisplatin was obtained from the UKSH dispensary. Spheroids were cultivated as described above. After 72 h of growth, half of the medium was replaced and the spheroids were treated with 100 µM cisplatin or PBS for an additional 24 h. Simultaneously with the medium change, the CellTox Green Assay (Promega G8731, Madison, WI) was added in a final dilution of 1:2000 to ensure good dye penetration. Cytotoxicity was determined with the CellTox Green Assay 24 h after treatment, using the automated cell imager NYONE Scientific (SYNENTEC, Elmshorn, DE) with 4× magnification objective. The following excitation sources and emission filters were used: Brightfield (BFEx/GreenEm (530/43 nm)); CellTox Green (BlueEx (475/28 nm)/GreenEm (530/43 nm)). For quantification, the fluorescence signal within the spheroid was analysed using the spheroid quantification (1 F) (v. 0.9) (2 channel) application in YT-Software (SYNENTEC, Elmshorn, DE) using the result “Average fluorescence intensity CH1 BC”.

24 h after treatment the endpoint was reached and viability and apoptosis were measured by RealTime-Glo (460_Em_) (Promega G9711, Madison, WI) and Caspase-Glo 3/7 (565_Em_) (Promega G8090, Madison, WI), using a microplate multimode reader (Spark, Tecan, Männedorf, Schweiz, CH). The measurement was performed according to the instructions measuring luminescence signal^71^. The viability values were used to normalize the caspase results (relative caspase activity: caspase activity divided by the viability (normalized to control)).

In another experiment, live-dead staining was performed. For this purpose, the spheroids were grown and treated as described above. Three hours before the end of treatment, the dyes propidium iodide (PI) (10 µg/mL), calcein-AM (1 mM), and Hoechst 33342 (0.001 %) were added to the spheroids. For imaging, the BZ-X810 (Keyence, Osaka, JPN) microscope with 10x magnification objective was used. Z-stack images of this spheroids were taken and merged. This analysis were all established in our group and performed as previously described^25–27^.

###  Statistical analysis and software

Statistical tests were performed using GraphPad Prism version 10.2.2 (GraphPad Software, San Diego, CA. The means of at least three replicates were calculated. Gaussian distribution was tested by the Shapiro–Wilk normality test. One-way analysis of variance (one-way ANOVA) and t-test was used to test for differences in means between multiple groups, followed by Dunnett multiple comparison procedure for pairwise comparisons. Statistically significant differences were assumed at *p*-values < 0.05 (*). BioRender.com. and Gimp 2.10.18 (https://download.gimp.org/gimp/v2.10/windows/) were used to create the images. YT-SOFTWARE (SYNENTEC, Elmshorn, DE), ZEN 3.5 (blue edition) (Carl Zeiss Microscopy, Oberkochen, DE), Keyence (BZ-X810, Osaka, JPN) were used for microscopy analysis. Spark and SparkControl (Tecan, Männedorf, Schweiz, CH) were used for fluorescence and luminescence quantification). Microsoft Windows 10 (Microsoft Corporation, Redmond, W) were used for data organization.

## Electronic supplementary material

Below is the link to the electronic supplementary material.

Supplementary Material 1. Supp Fig. 1. Compactness mono and co-cultured spheroids and Ki67 Index Supp Fig. 2. 2.5D LSM images of the midplane of the 3D spheroids.

## Data Availability

All data associated with this study are present in the paper. The data presented in this study are available on request from the corresponding author.
