# Supplementary material for: Multicellular ovarian cancer spheroids: novel 3D model to mimic tumour complexity
Source: Sci Rep. 2024 Oct 9;14:23526. doi: 10.1038/s41598-024-73680-6 (PMC11464915; doi:10.1038/s41598-024-73680-6)
Supplement: Supplementary file 1 — Supplementary Material 1. Supp Fig. 1. Compactness mono and co-cultured spheroids and Ki67 Index Supp Fig. 2. 2.5D LSM images of the midplane of the 3D spheroids. [file 41598_2024_73680_MOESM1_ESM.docx]

Supporting Information

Research Article

Multicellular ovarian cancer spheroids: Novel 3D model to mimic tumour complexity

Inken Flörkemeier ^1,4^*, Lisa K. Antons ^1^, Jörg P. Weimer ^1^, Nina Hedemann ^1^, Christoph Rogmans ^1^, Sandra Krüger ^2^, Regina Scherließ ^3,4^, Astrid Dempfle ^5^, Norbert Arnold ^1^, Nicolai Maass ^1^, and Dirk O. Bauerschlag ^1,6^

**
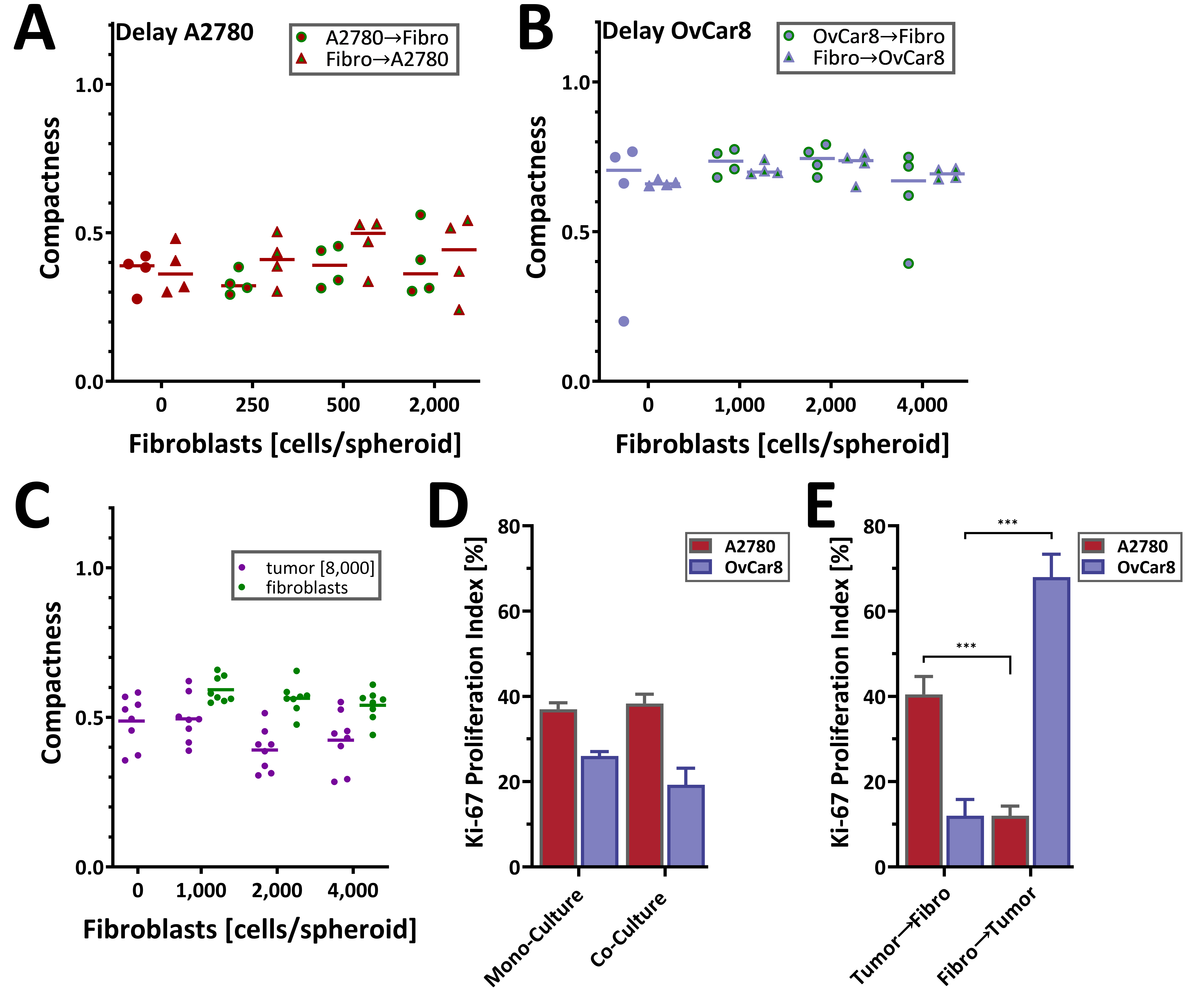
**

**Supp Figure 1. Compactness mono and co-cultured spheroids** **and Ki67 Index**. Spheroids were cultured in mono-culture and in co-culture with ovarian cancer cells and different cell numbers of fibroblasts for 96 h in ULA plates. (B) Ovarian cancer cell lines OvCar8/A2780 and fibroblasts (Detroit 551) were sequential seeding for 96 h. Compactness of the spheroid formation were determine. Quantitative data are means, N=3, t-test. (C) Ovarian cancer primary cells (UF-403) and fibroblasts (UF-403) were simultaneously seeding for 96 h. Compactness of the spheroid formation were observed. Quantitative data are means, N=3, one-way ANOVA. (D, E) Immunohistochemical analysis of spheroid morphology and protein expression of OvCar8 and A2780 spheroids after 96 h of growth were performed. The Ki-67 index after staining was calculated (D: simultaneous seeding of OvCar8 and A780 spheroids, E: sequential seeding of OvCar8 and A780 spheroids) Quantitative data are means, N=3, t-test *** (p <0.001).


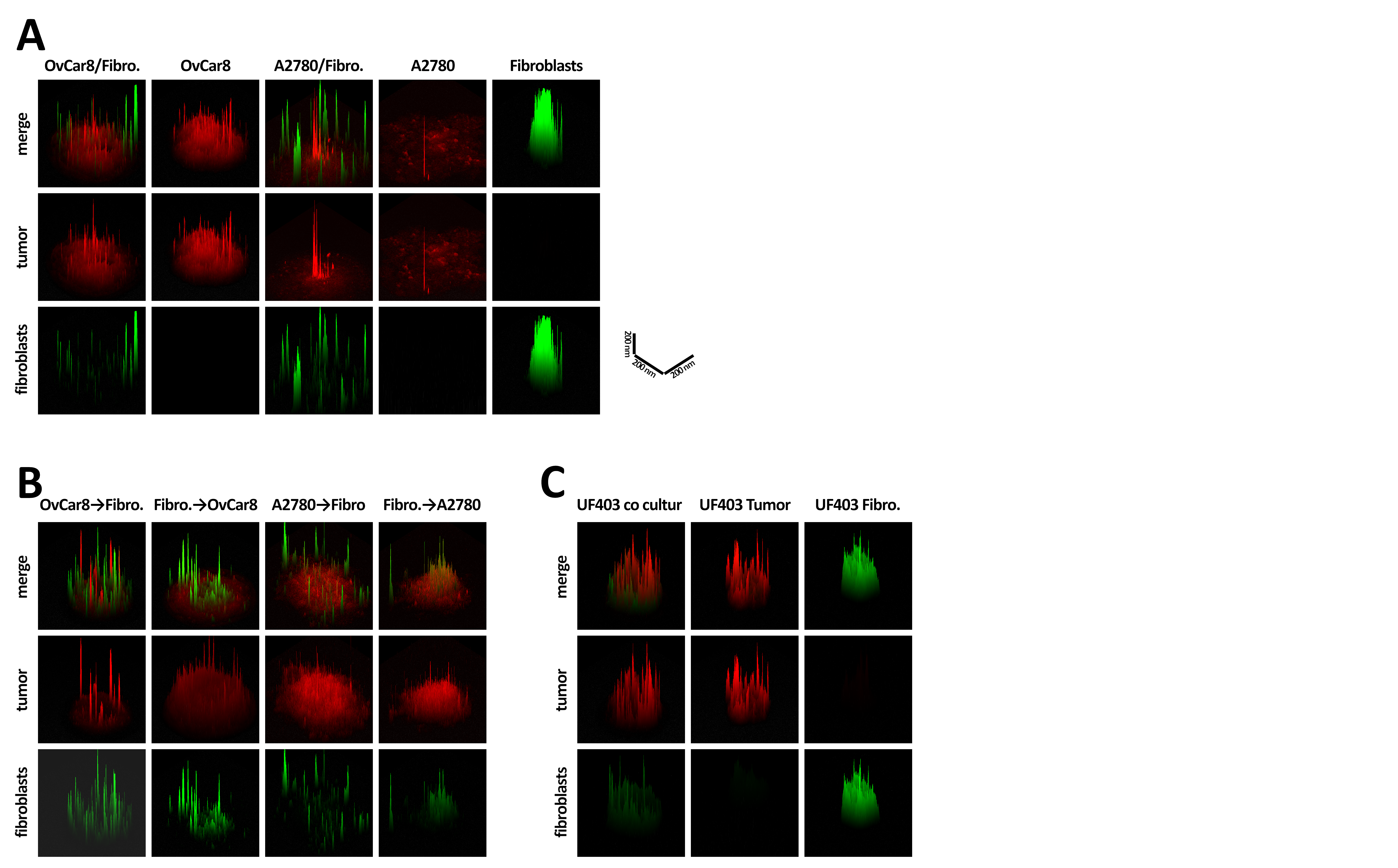


**Supp Figure 2. 2.5D LSM images of the midplane of the 3D spheroids.** Prior to cultivation, the cells were stained with fluorescence dyes in order to subsequently assign them to a cell type during cultivation. These spheroids were fixed, cleared and imaged after 96 h of growth using LSM. Red/Cell Tracker Deep Red: ovarian cancer cells; green/CellTracker Green CMFDA: fibroblasts. Scale bar 200 µm. (A) Simultaneous seeding of cancer cell lines OvCar8/A2780 and fibroblasts (Detroit 551). (B) Sequential seeding of cancer cell lines OvCar8/A2780 and fibroblasts (Detroit 551). (C) Simultaneous seeding of primary ovarian cancer cells (UF-403) and fibroblasts (UF-403).
